# From naturalness to materiality: reimagining philosophy of scientific classification

**DOI:** 10.1007/s13194-023-00509-w

**Published:** 2023-01-21

**Authors:** David Ludwig

**Affiliations:** Knowledge, Technology, and Innovation (KTI) Group, Wageningen University, Wageningen, Netherlands

**Keywords:** Natural Kinds, Classification, Materiality, Feminist Philosophy of Science

## Abstract

The notion of natural kinds has been widely criticized in philosophy of science but also appears indispensable for philosophical engagement with classificatory practices. Rather than addressing this tension through a new definition of “natural kind”, this article suggests materiality as a substitute for naturalness in philosophical debates about scientific classification. It is argued that a theory of material kinds provides an alternative and more inclusive entry point for analyzing classificatory practices, which is specified through an account of “restricted malleability” of materiality and further analyzed as (1) gradual, (2) multi-dimensional, (3) scalable, (4) interactive, and (5) purpose sensitive.

## Introduction

The debate about natural kinds appears to have reached an impasse. For decades, the “tradition of natural kinds” (Hacking, 1991) has been criticized by philosophers as obscuring the complexity of classificatory practices in science. The idea that scientists “carve nature at its joints” by identifying the essences of mind-independent and objective discontinuities in nature has been widely challenged by anti-essentialist and pluralist arguments (e.g. Chang, 2012; Dupré, 1993; Kitcher, 1984; Ludwig & Ruphy, 2021). While there is no shortage of attempts to reformulate the tradition of natural kinds, a growing number of philosophers have lost their patience with this reformism and have suggested addressing scientific classification without any appeal to natural kinds. For example, Chakravartty (2017, 78) argues that “theorizing about natural kinds, the philosophical study of categories of things in nature, should ultimately give way to scientific classification.” Reflecting on the heterogeneity of fruitful classifications in science and other domains, Ludwig (2018, 31) suggests “that attempts to formulate a general account of naturalness have become an obstacle to understanding classificatory practices.” Challenging the association of natural kinds with value-free objectivity (Ahn, 2021), Brigandt (2022, 1) argues “that philosophers should stop using the term ‘natural kinds,’ as this label obscures the relevance of humans interests and the way in which many kinds are based on contingent social processes subject to human responsibility.”

Despite this growing chorus of complaints, the notion of natural kinds has proven to be remarkably resilient in the literature. There is a constant stream of new proposals on how to define “natural kind” (Barberousse et al., 2020; Ereshefsky, 2018; Ereshefsky & Reydon, 2019; Ferreira Ruiz & Umerez, 2018; Franklin-Hall, 2015; Khalidi, 2015a; Lemeire, 2020; Magnus, 2012; Slater, 2015; Tahko, 2022), reflecting a prevailing sentiment that the tradition of natural kinds remains indispensable for analyzing classificatory practices. Even philosophers who are critical of the current state of natural kind debates commonly embrace reformism rather than eliminativism. For example, Ereshefsky and Reydon (2019, 34) argue that we should remain “optimistic about the usefulness of ‘natural kind’ as a philosophical concept” and Conix and Chi (2021) argue that the elimination of “natural kind” would amount to the elimination of a useful entry point for philosophical and interdisciplinary debates about scientific classification.

The aim of this article is to move beyond this impasse by developing an account of material kinds as an alternative to patching holes in the tradition of natural kinds. While such a proposal eliminates “natural kind” in favor of a much more inclusive notion of “material kind”, the specification of materiality also conserves important insights from debates about naturalness such as the interplay between conceptual decisions and empirically discovered structures of the material world. A theory of material kinds is eliminativist in the sense that it is not only letting go of the term “natural kind” but also abandons the project of identifying a natural “elite” (Lewis, 1983) that is clearly distinguished from ubiquitous non-natural kinds. At the same time, it is substitutionist in the sense that it preserves substantial philosophical insights that has been traditionally associated with the tradition of natural kinds: explaining how kinds differ in epistemic features such as projectibility, showing how they are grounded in material features such as property clustering, and distinguishing epistemically fruitful kinds from mere linguistic constructions without material grounding.

The first part of the article (Sect. 2 and 3) addresses recent attempts to save the notion of natural kinds. While these attempts fail to overcome core problems of the tradition of natural kinds, I argue that they identify an important shortcoming of natural kind eliminativism by highlighting the need for an overarching theoretical framework for engaging with classificatory practices (Sect. 4). The second part of the article develops an account of material kinds as an inclusive alternative to natural kinds (Sects. 5 and 6). Shifting the debate from “naturalness” to “materiality” redirects the focus from boundary disputes about naturalness to the “restricted malleability” of material kinds that is further specified as (1) gradual, (2) multi-dimensional, (3) scalable, (4) interactive, and (5) purpose sensitive.

## Saving Natural Kinds: The Naturalistic Strategy

Much of the current debate about scientific classification is sensitive to shortcomings of the tradition of natural kinds such as excessive essentialism, monism, and claims of mind-independence. Indeed, there would be little hope for “natural kind” if the notion was tied to the assumption that scientific classification is always based on the discovery of structural essences such as H_2_0 that demarcate the boundaries of natural kinds such as water independently of contingent aims and values. Many scientific kinds lack structural essences and it has become widely recognized that science comes with many classificatory practices that are far from mind-independent (Ereshefsky, 2018; Khalidi, 2016) and navigated on the basis of epistemic and non-epistemic aims of scientists (Brigandt, 2022; Ludwig, 2016). In fact, traditional framings of natural kinds have been challenged even in their application to classical examples such as water and H_2_0 (Chang, 2012).

This critique, however, does not imply that scientific classification is a free-floating exercise of worldmaking and many philosophers highlight the need to reform the tradition of natural kinds without excessive demands of essentialism, monism, and mind-independence. At the same time, the success of this reformism remains contested as it runs the risk of turning “natural kind” into an ambiguous and vague notion that creates more confusion than clarity (Hacking, 2007). Ludwig (2018) argues that the current literature on natural kinds identifies many different “dimensions of non-arbitrariness” and descends into scholastic priority disputes about declaring one of them as the demarcation criterion for naturalness. For example, Boyd (1999) has pointed out that many categories such as biological species or chemical elements or minerals come with clusters of correlated properties that are stabilized through homeostatic mechanisms. Slater (2015) has argued that there is a broader phenomenon of stable clustering of properties that does not always require homeostatic mechanisms. Franklin-Hall (2015) has pointed out that some categories constitute “categorical bottlenecks” in the sense that they can serve heterogeneous epistemic aims. And so on.

Once these different dimensions from homeostatic mechanisms to stable property clustering to epistemic fruitfulness are understood, however, the notion of natural kinds is not only expendable but risks creating artificial boundary disputes about the naturalness of categories. For example, consider a category that satisfies Slater’s (2015) characterization of a stable property cluster but not Franklin-Hall’s (2015) characterization of categorical bottlenecks because the cluster is interesting only for highly specific epistemic purposes. Should we accept that such a category is identifying a natural kind? According to Ludwig (2018), such a boundary dispute is best avoided by giving up on the notion of natural kind. We can simply acknowledge that a category identifies a stable property cluster in the sense of Slater but does not constitute a categorical bottleneck in the sense of Franklin-Hall without ever worrying about the question which dimension should define naturalness.

In a recent series of articles, Ereshefsky and Reydon (2019) propose an answer to this eliminativist challenge. Ereshefsky and Reydon agree with much of Ludwig’s discontent about the current state of natural kind debates and suggest that it is “too divorced from actual classificatory practice to be relevant to that practice” (2019, 4). At the same time, they argue that the problem is not the notion “natural kind” but rather that attempts to explicate it have been “insufficiently naturalistic” as they do not track the actual uses of the notion in science and philosophy. In response, Ereshefsky and Reydon propose a “grounded functionality account” that defines the naturalness of a kind through two necessary and jointly sufficient conditions: First, a natural kind needs to be functional in the sense that it satisfies whatever epistemic or non-epistemic aims of a classificatory program. Second, this functionality needs to be achieved through grounding in the world. Natural kinds are anchored in the empirical world and their functionality is based on this anchoring.

However, there is a clear tension between Ereshefsky and Reydon’s case for a naturalist methodology and their ambition to formulate a unified account of natural kinds. The reality is that scientists appeal to naturalness in highly heterogeneous ways and a naturalist methodology therefore casts doubt on the feasibility of any conceptual unification. Indeed, the grounded functionality account is naturalistic enough to capture at least some uses of “natural kind” in the empirical literature. Just as its competitors, however, the grounded functionality account is not thoroughly naturalistic in the sense of capturing the heterogeneous meaning of “naturalness” in the empirical literature. Indeed, grounded functionality captures what *some* “philosophers and scientists aspire to when they talk about *natural* classifications'' (2019, 32). But it certainly does not capture the ambitions of *all* philosophers and scientists. Appeals to naturalness in the empirical literature can serve both broader and more limited ambitions than identifying all categories that satisfy conditions of grounded functionality.

As Ereshefsky and Reydon aim to develop an inclusive account of natural kinds, it is not surprising that talk about natural kinds in the empirical literature often does not fit their proposal because it reserves “naturalness” for something more specific than “grounded functionality”. For example, consider Ludwig’s (2018) case of ethnobiology. Much of the ethnobiological interest in natural kinds has been focused on a rather restrictive subset of categories that are cross-culturally stable and contrast with merely locally recognized categories and property clusters. In this context, the aspiration of natural kind talk is to account for categories that are grounded in ways that heterogeneous cultures in geographically disconnected locations recognize them in similar ways (Atran, 1981; Berlin, 1992; Ellen, 2003). This aspiration in the ethnobiological literature fits very well with Franklin-Hall’s (2015) account of natural kinds as “categorical bottlenecks” that converge because they serve the interests of heterogeneous epistemic actors. It does decisively not fit the grounded functionality account that allows for natural kinds that serve the aims of very specific classificatory programs rather than restricting the notion to those that serve a variety of unrelated programs. While there may be good reasons to adopt such a more inclusive proposal, it does not follow a thoroughly naturalistic approach as many scientific practices restrict talk about naturalness to something more specific such as categorical bottlenecks.

Of course, this is not to say that appeals to naturalness in the empirical literature are always restricted to categorical bottlenecks. On the contrary, the challenge for any general account of natural kind is that appeals to naturalness express heterogenous ambitions. In ethnobiology, for example, an exclusive focus on categorical bottlenecks will miss biological kinds that are recognized only by specific communities because they are only relevant for very specific aims or only stable in very specific environments (Ludwig, 2017, 2018). In such cases, appeals to naturalness are indeed better captured by more permissive accounts such as Ereshefsky and Reydon’s grounded functionality or Slater’s stable property clusters. However, even grounded functionality may sometimes turn out to be too restrictive. Discussing ethnobiological classification, for example, Reydon and Ereshefsky argue that a folk classification of whales as fish could be grounded in the world through shared habitat and similar ways of living but would still not qualify as a natural kind “as long as the relevant community does not provide a well-confirmed account that connects” the aims of this classification with aspects of the biological world (2022, 13).

Restricting “natural kind” in this way may serve Ereshefsky and Reydon’s goals of understanding classificatory programs in scientific practice. It is too narrow for many ethnobiologists whose primary goal is not to understand scientific practice and who are sensitive to the—epistemic but also political—risks of equating natural and scientific kinds. For many ethnobiologists, it is relevant whether community categories support local practices and livelihoods by capturing relevant property clusters in the biological world (Robles-Pineros et al., 2020) rather than to demand that communities have developed a “classificatory program [that] provides a well-supported account of how the Functionality and Grounding Conditions are met” (Reydon & Ereshefsky, 2022, 13). This is not to say that Ereshefsky and Reydon are wrong to demand such an account in the context of their particular interests as philosophers of science but it is less plausible in the context of ethnobiologists trying to understand community classifications. There is nothing wrong with the grounded functionality account but it is shaped by its own contingent ambitions rather than somehow capturing the ambitions of naturalness talk across philosophy and science.

While the grounded functionality account identifies an interesting set of kinds, positioning it as a unified account of “natural kind” therefore runs into exactly the same problem as all of its competitors: It nicely captures some talk about “natural kinds”. It is too liberal to capture other talk about “natural kinds”—for example when the notion is restricted to categorical bottlenecks that are recognized by heterogeneous epistemic actors. It is too restrictive to capture even other talk about “natural kinds”—for example when the notion is expanded to categories that identify stable property clusters but are not associated with a classificatory program. Just as its competitors, grounded functionality captures some but not all talk about “natural kind” in the empirical literature. This is not a flaw in itself but becomes a problem once grounded functionality is positioned as a unified account of “natural kind” across all scientific and philosophical contexts. As the account prioritizes some ambitions over others, using it for a general definition of naturalness will inevitably generate scholastic boundary disputes with scholars who have other ambitions and prefer to draw the boundaries of natural kind differently.

A non-naturalist strategy may respond to this situation by trying to discipline scientists and philosophers in their use of “natural kind”—maybe everyone should always use “natural kind” in the sense of grounded functionality and should stop appealing to naturalness in other senses such as categorical bottlenecks or stable property clustering. However, such a revisionism would require that Ereshefsky and Reydon’s give up on their naturalist commitments. A naturalist methodology that does not embrace revisionism therefore reinforces the case against any unified account of naturalness or natural kind as scientists do not express one unified ambition with these notions.

## Saving Natural Kinds: The Pragmatic Strategy

Both Hacking and Ludwig argue that the tradition of natural kinds is a mess and that the prospects of unification are bleak. While Ereshefsky and Reydon (2019) respond to this challenge with a new unificationist proposal, Conix and Chi (2021, 3) develop a defense of “natural kind” that accepts its messy state and recommends to embrace it: “there are many different kinds of kinds, and there is no single theory of natural kinds that can account for all of them”. In fact, Conix and Chi even accept Ludwig’s (2018) suggestion that “natural kind” plays no productive theoretical role and that the elimination of “natural kind” would not lead to any loss of explanatory power as philosophers could simply talk about more specific phenomena such as the clustering of properties or issues of projectibility and epistemic fruitfulness without ever appealing to a general account of “natural kind”. However, Conix and Chi suggest that “natural kind” still plays a productive pragmatic role in linking these proposals and enabling a fruitful research debate about classificatory practice.

As Conix and Chi admit, this pragmatic argument leads into rather fuzzy territory as it needs to show that the benefits of conserving “natural kind” somehow outweigh the benefits of eliminating the term. In developing their case for the conservation of “natural kind”, Conix and Chi mainly rely on bibliometric evidence of the use of “natural kind” in both philosophical and interdisciplinary literature. First, they present an analysis of co-citation patterns of natural kind articles in leading philosophy journals. Their evidence demonstrates a well-integrated author network that indicates active intellectual exchange through common reference literature about natural kinds. Conix and Chi suggest that this result supports the assumption of an important pragmatic function of “natural kind” in providing a shared entry point for philosophical debates about kinds and classification.

While Conix and Chi’s data indeed show that “natural kind” has a pragmatic function in the literature, it is much less clear that they demonstrate an *epistemically fruitful* function. Natural kind eliminativists like Hacking (2007) and Ludwig (2018) do not deny that the tradition of natural kinds provides a popular entry point for philosophical debates about classification and in this sense has a pragmatic function of creating a shared entry point. However, they consider “natural kind” an epistemically detrimental entry point that does not foster philosophical understanding of scientific classifications but instead encourages a scholastic and self-referential debate. While Conix and Chi treat co-citation patterns as indicators of epistemic fruitfulness they may as well be treated as symptoms of the self-referential scholasticism that Hacking and Ludwig are warning about. Indeed, this is a familiar phenomenon from other debates that have fallen out of favor within academic philosophy such as definitional debates about “knowledge” as “justified true belief” following Gettier (1963). While Gettier’s thought experiment created a cottage industry of closely connected articles with a tightly knit network of co-citations, the focus on competing definitions of “knowledge” has been criticized as leading to an overly narrow perspective on epistemology (Beckermann, 2001). Conix and Chi’s data on co-citation patterns seem equally compatible with epistemologically optimistic and pessimistic diagnoses: they may be an indicator of an epistemically fruitful research debate or a symptom of a self-referential scholastic discourse.

Conix and Chi are aware of these limitations and present a second analysis of connections between philosophical and empirical literature. If “natural kind” plays an important role in connecting philosophical and empirical research on classification, the charge of self-referential scholasticism seems unfair. And indeed, their data suggest that “natural kind” constitutes an interdisciplinary entry point for debates about scientific classification. This result challenges some of the overly dismissive comments by eliminativists and suggests that simply “letting go of natural kind” (Ludwig, 2018) may do more harm than good by removing a shared notion for empirical and philosophical debates about scientific classification.

While Conix and Chi highlight the importance of an entry point for debates about scientific classifications, their argument only challenges a straightforward eliminativism that does not propose any alternative. Conix and Chi do not (and do not claim to) show that “natural kind” provides the *best available* or even the *best possible* entry point for debates about scientific classification. Their argument does not challenge arguments that the tradition of natural kinds constitutes an epistemically problematic entry point that encourages unproductive definitional disputes as argued by Ludwig (2018) or obscures the role of human purposes in scientific classification as argued by Brigandt (2022). Instead, their evidence suggests that conserving “natural kind” is better than having no entry point whatsoever. In this sense, their pragmatic argument challenges critics of the tradition of natural kinds to show that we can do better by providing an epistemically more productive entry point for debates about classificatory practices. The following sections will argue that we can indeed do better by shifting our attention from naturalness to materiality.

## Conceptual Liberation and Poststructuralist Disappointments: The Return of Materiality

Even if the tradition of natural kinds fails to identify one unified phenomenon, it engages with a range of phenomena that are of utmost importance for understanding classificatory practices. For example, some categories are more successful in supporting inductive generalizations than others. Some categories refer to more stable clustering of properties than others. Some categories are more unified in terms of underlying causal structures than others. Some categories are cross-culturally and cross-disciplinarily more stable than others. Even if natural kind eliminativists like Hacking and Ludwig are correct that these phenomena do not reduce to one unified phenomenon that can be captured through a general definition of “natural kind”, any epistemically fruitful philosophy of classification needs to provide resources for engaging with these entangled phenomena.

One reason to be suspicious of natural kind eliminativism is the worry that it will leave philosophical debates without resources for engaging with these issues. Even if the tradition of natural kinds is in bad shape, it provides at least some entry point. This section shifts from philosophy of science to the recent history of feminist philosophy to develop a negative and a positive claim. First, the rise and fall of poststructuralist accounts of categories such as *gender* and *sex* illustrates the risks of eliminating the tradition of natural kinds without a clear alternative for engaging with material structures and processes. Second, materialist approaches in feminist philosophy are introduced as a promising starting point for articulating an alternative entry point for debates about classification that avoids the pathologies of both the natural kind tradition and poststructuralist philosophies of classification.

It is helpful to contrast the more recent criticism of “natural kind” in philosophy of science (Brigandt, 2022; Chakravartty, 2017; Hacking, 2007; Ludwig, 2018) with decades of poststructuralist critique of appeals to nature and naturalness. In poststructuralist debates, eschewing naturalness has often been framed as a form of conceptual liberation that opens spaces for negotiating contested categories such as *gender* and *sex* by allowing “the interrogation of these formations as fundamentally social, precisely because dominant essentialist accounts legitimized gender/sexual inequalities as ‘natural’ and thus inevitable and immutable” (Rahman & Witz, 2003, 244).

Narratives of conceptual liberation from essentialism by philosophers like Butler (1990, 1993) and Foucault (1971, 1976) have successfully captured the imagination of diverse audiences, but poststructuralist frameworks come with their own limitations and disappointments. While circumventing pathologies of the tradition of natural kinds such as the depolitization of classificatory practices, there has also been a growing sense that poststructuralism created its own pathologies by failing to provide positive frameworks for analyzing categories beyond discourse and power. Categories such as *gender* and *sex* (or *disability, homosexuality, mental disorder, race,* etc.) involve contingent and political elements, but they do not reduce to discursive politics. For example, the tradition of natural kinds may have obscured the political dimensions and contingencies of many psychiatric categories and of the more general question of what counts as a mental disorder in the first place (Zachar, 2014). However, the politics of psychiatric classification does not imply that categories of mental disorders are *nothing but* arbitrary conventions for discursive politics. Instead, the political dimensions interact with social, psychological, and biological mechanisms that need to be studied carefully in any critically reflective psychiatric practice. Substituting the tradition of natural kinds entirely with discursive power seems to trade one set of pathologies for another.

In feminist philosophy, much of the discontent with the limitations of poststructuralism has become expressed in a return of materiality and materialism. For example, Jagger (2015, 321) introduces the “new materialism” in feminist philosophy as “a response to the linguistic turn that has dominated the humanities in the past few decades and that, it is claimed, has neglected the materiality of matter.” Jackson (2001, 285) takes more explicit aim at poststructuralist philosophies by arguing that their project of subversive conceptual liberation ultimately made them “lose touch with material social structures and practices. It became impossible to think of ‘women’ and ‘men’ as social categories, products of a structural hierarchy […]. The cultural turn effectively sidelined this materialist analysis and emptied the concept of gender of its social import as a hierarchical division between women and men.”

The materialist turn in feminist theory is a promising starting point because it is not a return to the tradition of natural kinds and its tendency to depoliticize classifications as value-free practices of “carving nature at its joints.” The debate about materiality in feminist theory has complex genealogies (Rahman & Witz, 2003)—it reflects feminist engagment with the biological sciences and the human body, with Neo-Marxist historical materialism and social structures, and with the challenges of Global South feminism that shifts the focus from conceptual liberation to “material effects of a particular imperial power structure [and] a materialist analysis that linked everyday life and local gendered contexts and ideologies to the larger, transnational political and economic structures and ideologies of capitalism” (Mohanty, 2003).

With the rise of poststructuralism in feminist theory, these overtly political angles of historical materialism converged with a distinctively metaphysical worry. An exclusive focus on discourse rather than material mechanisms of oppression became not only politically criticized as prioritizing bourgeoise concerns of white urban feminism but also metaphysically challenged as dissolving bodily and social materiality into discourse. As Butler (1993, 28) puts the common objection: “If everything is discourse, what happens to the body? If everything is a text, what about violence and bodily injury? Does anything matter in or for poststructuralism?”.

Butler (1993) responds to this objection with a performative account of materiality that is far more complex than the self-serving caricature of poststructuralism as linguistic idealism in which materiality becomes reduced to discourse. At the same time, poststructuralist frameworks often remain unsatisfying in practical engagement with the material world because they do not provide much more than a generic and vague commitment to materiality which is specified only through a shift back to discursive practice. As Karen Barad puts it: “Questions about the material nature of discursive practices seem to hang in the air like the persistent smile of the Cheshire cat” (2007, 64). Furthermore: “What is needed is a robust account of the materialization of all bodies […] including the agential contributions of all material forces (both ‘social’ and ‘natural’).” (2007, 64).

The recent history of feminist philosophy provides a wealth of theoretical resources for developing accounts of materialization. Unsurprisingly, much of feminist philosophy has focused on the materiality of the human body as a site of feminist politics and of scientific investigation in biological, medical, and social sciences. However, the body does not return as a natural kind or even as a biological essence but rather as a material actor that shapes social and political practices as much as it is shaped by them. Materiality is not simply a new term for naturalness but can be interpreted as an attempt to overcome the shortcomings of both traditions of natural kinds and poststructuralism through an interactionist model of mutual co-constitution of material and classificatory practices (Barad, 2007).

While much more could be said about the state of materialism in feminist philosophy, the present sketch is sufficient to formulate two lessons for controversies about natural kinds in philosophy of science. First, there is a negative lesson about the risk of eliminating “natural kind” without a substantial alternative in place. While poststructuralist approaches successfully challenged the tendency to depoliticize contested categories in the tradition of natural kinds, they also run the risk of obscuring dimensions of scientific classification that do not reduce to discursive politics. This first part of the story therefore complements Conix and Chi’s (2021, 6) pragmatic case for “natural kind” as an “investigative entry into classificatory practices in science.” Second, recent debates about materiality suggest an alternative entry point with the potential to avoid at least some of the pathologies of the traditions of natural kinds and postructuralism. Conserving “natural kind” may be justified if it is without alternative in debates about classificatory practices. However, a move from naturalness to materiality may provide such an alternative.

## Towards a Theory of Material Kinds

The previous section suggested a shift from naturalness to materiality in analyzing classificatory practices. This shift is not merely terminological and the aim of this section is to show how materiality provides a novel and fruitful entry point for thinking about kinds. Most strikingly, materiality contrasts with naturalness through its inclusivity—one may wonder whether *planet* or *vegetable* or *airplane* is a natural kind but there is little point in wondering whether it is a material kind. Inclusivity is especially salient for materiality in the feminist tradition that has been shaped by concerns about both the materiality of the body and the materiality of social reality. While materialism in philosophy of science tends to be associated with the “scientific materialism” of the former, feminist perspectives tend to put at least equal emphasis on the “historical materialism” of the latter. Social kinds like gender are not merely discursive or symbolic constructions but materially constituted through social mechanisms, institutions, and practices that are accessible through empirical research.

Material kinds conceived through the feminist tradition are therefore permissive or “easy” (Thomasson, 2014) in the sense that they include most kinds of natural sciences, social sciences and everyday life. This is not to say that every kind is a material kind. For example, categories can fail to identify any real material structures. Think of a failed scientific field such as phrenology. As the shift from naturalness to materiality increases permissiveness, some talk about cranial areas will still identify material kinds—the cranium is real and so are at least some of the anatomical and morphological features that phrenologists described. While some cranial areas will be real material kinds, they will also be rather superficial kinds without much predictive or otherwise epistemic function. People with diverse cranial features are as real as people with differently shaped ear lobes, there just is not a whole lot to learn from it. While anatomically or morphologically defined cranial areas may still qualify as material kinds, other phrenological categories will fail to identify anything real in the material world. Think of a cranial area that is not demarcated anatomically or morphologically but rather as the seat of “superior sentiments” such as benevolence or wit or wonder. Such cranial areas simply do not exist and categories such as *cranial area for benevolence* therefore fail to identify any material kinds.

The example of phrenology provides two lessons about the move from natural to material kinds. First, the permissiveness of materiality compared to naturalness does not imply that everything becomes a material kind. Some categories simply do not identify anything real in the material world. Second, the permissiveness of materiality also means a shift away from boundary work as the main intellectual tool for understanding scientific classifications. In the tradition of natural kinds, the understanding of scientific classifications is closely tied to the project of separating a restricted natural “elite” (Lewis, 1983) from the remainder of common non-natural kinds. In contrast, kinds in successful scientific practice are often trivially material and trivial kinds such as anatomically defined cranial areas are still often material. The fruitfulness of a permissive account of material kinds is therefore not derived from boundary work but rather from analyzing the material constitution of kinds. In other words: the interesting philosophical question is usually not whether x is a material kind (in analogy to the question whether x is a natural kind) but rather in what ways x is materially constituted.

The starting point of such an analysis is a dynamic of what I want to call *restricted malleability.* Material kinds are shaped by intervention, in this sense they are inherently malleable. At the same time the malleability of material kinds is not unrestricted—you can’t create everything out of anything. The notion of restricted malleability therefore navigates between the marginalization of agency in the tradition of natural kinds that focuses on empirically discovered “joints in nature” and the marginalization of materiality in poststructuralist traditions that emphasize linguistic construction.

Restricted malleability relates to the interplay of conceptual and non-conceptual dimensions but does not reduce to them. Indeed, there are some cases such as the boundaries of *planet* where malleability falls entirely on the conceptual side (e.g. we can define *planet* in ways that include or exclude Pluto) without affecting the material side (e.g. one way or another we’re not changing the material structure of Pluto). In other cases, however, malleability/restriction does not simply map onto the conceptual/material. Feminist debates about classifications of bodies highlight malleability of the material rather than only the conceptual side as it is not only concepts of bodies but also bodies themselves that are transformed through a wide range of social practices from sports to hormone therapies to dietary choices to plastic surgery to makeup choices to psychosomatic feedbacks.

Just as malleability is not exclusive to the conceptual side, restriction is not limited to the material side. In ethnobiology, for example, it has been commonly argued that cross-cultural variability of bioontologies is restricted by general features of cognitive and linguistic processing rather than merely the material structure of the target domain (Atran and Medin, 2008). The scope of such restrictions itself remains contested—some are widely accepted (e.g. societies without written language will restrict the number of biological kinds in use) while others remain contested (e.g. the assumption of a folk-biological module that restricts biological cognition of humans). Other cases of restriction on the linguistic side come from the pragmatic interplay between empirical detail and applicability—e.g. psychiatric kinds are not only restricted by the structure of the target domain but also by the need to formulate classifications that are not overly complex for applicability in psychiatric practice. The emphasis on restricted malleability therefore interacts but does not reduce to the material and conceptual side of debates about kinds (Table 1).

**Table 1:** Note: This data is mandatory. Please provide

	*Material*	*Conceptual*
*Malleability*	Material structures (e.g. properties and their clustering) are shaped through intervention	Classifications are shaped by variable epistemic and non-epistemic interests into material structures
*Restriction*	Material structures (e.g. properties and their clustering) limit the range of classificatory options	Classifications are limited by (e.g. cognitive or pragmatic) demands towards linguistic representation

Taking restricted malleability as a starting point allows for a nuanced approach to kinds that can be further specified through different features of material kinds as (1) gradual, (2) multi-dimensional, (3) scalable, (4) interactive, and (5) purpose sensitive.

### Graduality

One core feature of malleability is that it is gradual—some material kinds are more malleable than others. The tradition of natural kinds is often driven by what Franklin-Hall (2015, 926) calls the ‘‘very basic intuition’’ that there is a difference between “categories offered up by the mature sciences (e.g., *bosons, C. elegans*) and those not in scientific circulation—from the wildly pathological (e.g., *animals born on a Tuesday*) […], or the scientifically defunct (e.g., *consumption, hysteria*), to the merely boring.” Even if this intuition motivates much of the literature on natural kinds (see also MacLeod & Reydon, 2013, 91), it raises a rather straightforward concern: what about all the categories that are not part of “mature sciences” but also not pathological/defunct/boring? For example, what about categories of emerging sciences? What about categories of the humanities? What about contested categories such as *biodiversity, disability,* or *gender* that clearly play important roles in scientific practice but are not nearly as stable as *boson* and *C. elegans*? What about our everyday categories of different kinds of literature, music, machines, or garments that are far from pathological/defunct/boring even if they do not feature in “mature sciences”?

The tradition of natural kinds has a difficult relationship with these in-between spaces that do not neatly fall on either side of the demarcation line between carefully selected examples of natural and non-natural kinds. Often, they are simply ignored. Sometimes, they are dismissed as non-natural. Sometimes, the boundaries of the natural are redefined to include at least some of them. Shifting the attention from naturalness to materiality avoids these difficulties and the intellectual bias towards “mature sciences.” For example, categories of mature sciences (e.g. of biological species) and of everyday practices (e.g. of types of furniture) both clearly identify material kinds in the sense that they refer to material structures and are restricted by them.

Differences do not come in the form of a dichotomy between the maturely natural and the arbitrarily non-natural but through different degrees of malleability. Classifications of furniture, for example, show a relatively high degree of malleability. While this is far from a case of “anything goes” (try to convince someone that a strawberry is a piece of furniture but a table isn’t), different cultural practices of household organization lead to heterogeneous practices of classifying material objects as furniture. In comparison, biological species show a lesser degree of malleability. While debates about species pluralism have shown that boundaries of species are indeed malleable, it would be a misunderstanding to think that there are no relevant differences between species and furniture. For example, classifications of species are sometimes strikingly consistent across cultural contexts because they reflect phylogenetic relations that come with a tightly knit cluster of (e.g. behavioral, ecological, genetic, morphological) properties that is salient from different perspectives (Ludwig, 2017). Addressing different degrees of malleability can help to distinguish between different characteristics of kinds without having to categorize them as either maturely natural or arbitrarily non-natural.

### Multi-dimensionality

Material kinds are not only malleable to different degrees but also along different dimensions. Some categories are shaped along underlying physical or chemical structures. Other categories primarily reflect visible features or the functions of objects without assuming shared underlying structures. Other categories reflect shared histories such as phylogenetic relations. One persistent problem with the tradition of natural kinds is that it struggles with this multi-dimensionality while trying to formulate one general criterion for naturalness. For example, essentialist requirements of underlying chemical or physical structures have been largely abandoned in the literature on natural kinds as they exclude far too many successful scientific kinds. More liberal frameworks appeal to “homeostatic property clusters” (Boyd, 1999) and have become widely embraced because they can account for a larger variety of kinds. However, as Ereshefsky and Reydon (2015) point out, they can still obscure successful scientific categories that are not grounded in homeostatic mechanisms such as species concepts in microbiology or functionally defined gene concepts.

Shifting the debate from naturalness to materiality avoids this struggle of trying to formulate an account that is inclusive enough to capture the different dimensions of non-arbitrary kinds while still restrictive enough to capture the actual use of “natural kind” in the empirical literature (see Sect. 2). Without this baggage, there is no need to squeeze all the different (e.g. structural, morphological, historical, functional) dimensions of malleability into one general definition. All of the mentioned kinds—from biological species to furniture—are unproblematic examples of material kinds. While materiality is inclusive, there is room for a more nuanced analysis as material kinds differ from each other not only in different degrees but also in different dimensions of malleability. A theory of material kinds can focus on specifying these different dimensions and the relations between them without having to integrate them into a general definition of natural kind.

### Scalability

Material kinds can be found across different levels of organization. Atoms are material but so are molecules, cells, organs, organisms, artifacts, social institutions, or planets. While there can be little doubt about the scalability of material kinds, the tradition of natural kinds has a remarkably awkward relation with higher levels of organization and especially the social world. The early phase of the tradition of natural kinds avoided these problems by largely ignoring kinds of the social sciences. If “*natural* kind” is exclusively applied to *natural* sciences, there may be little reason to worry about issues of scalability. Many recent accounts of natural kinds, however, insist that their frameworks can also be applied to the social sciences rather than being restricted to natural sciences (Khalidi, 2015b).

This upscaling of natural kinds into the social sciences tends to be a constant source of confusion and misunderstanding. First, the rhetoric of naturalness creates clear tensions in its application to social kinds such as social groups or social institutions that would not be considered “natural” in any non-technical sense of the word. However, the problem reaches beyond mere terminological awkwardness of framing the social as natural. Consider the burgeoning literature on metaphysics of race. A substantial part of this literature has been focused on arguing that races are not natural kinds in order to reject the priority of biological perspectives of even biological essences that would suggest one objective racial division of the human species. Given the political risks of essentialist misunderstandings of racial categories, it is not surprising that it has become a mantra in the literature to emphasize that “race is not a natural kind” (e.g. Botts, 2016; Yancy, 2019; Nanay, 2010; Pierce, 2014). Much of the recent literature on natural kinds, however, proposes general demarcation criteria (homeostatic/stable property clusters, nodes in causal networks, categorical bottlenecks, grounded functionality, etc.) that can be satisfied by social kinds and may lead to the conclusion that race is in fact both a natural and social kind. At the same time, such a claim about the naturalness of human races invites misunderstandings by anyone who is not familiar with technical uses of “naturalness” that do actually not prioritize the natural sciences.

As highlighted in the previous section, controversies about gender provide another illustration of this dynamic. Again, it is commonly emphasized that gender is not a natural kind in order to point out that gender is not a simple expression of underlying biological structures and requires social science research rather than crude biological reductionism. While accounts of gender as a natural kind run the risk of being misunderstood, the legacy of poststructuralist feminism illustrates that claims about the “non-naturalness” of gender can also be misleading by suggesting arbitrary linguistic constructions that are disassociated from material realities.

Shifting from naturalness to materiality provides resources for avoiding such confusions. Calling gender or race a material kind rather than a natural kind does not suggest priority of the natural sciences or even some form of essentialism about them. The social world is material as well as especially salient in the context of feminist materialism that has opened spaces for talking about biology but also remains deeply shaped by historical materialism and its emphasis on the materiality of social institutions and practices. At the same time, emphasis on the materiality of gender and race also avoids association with arbitrariness of “non-natural kinds” and therefore provides a more solid entry point for articulating kind realism across scales of organization.

### Interactivity

One of the core differences between feminist materialism and the tradition of natural kinds is that the former puts emphasis on the interactivity of materials while the latter has often focused on discontinuities that are assumed to be simply discovered in nature and are therefore independent of human action. As Bird and Tobin (2017) put it in the opening paragraph of their article on natural kinds in the *Stanford Encyclopedia of Philosophy:* “To say that a kind is natural is to say that it corresponds to a grouping that reflects the structure of the natural world rather than the interests and actions of human beings.” In contrast, to say that a kind is material is not at all to say that it reflects structure independently of the interests and actions of human beings. Materials are not only malleable in different degrees, dimensions, and scales but often embedded in interactive processes that create complex causal networks and feedback loops between material structures and human actions. Think of gendered bodies as the most prominent example in the literature of feminist materialism. Human bodies are not merely natural objects that are described but they interact with our material practices from everyday routines to interventions that explicitly aim at reshaping bodies (e.g. diets, hormone therapies, sports, surgeries). Furthermore, material and discursive practices are interlinked in the engagement with bodies. As Barad argues, "linkage between discursive practices and their materializing effects on bodies is not at all mysterious; discursive practices are materially efficacious, to the extent that they are, because there is a causal linkage between them” (2007, 211). Thinking about the materiality of the body has therefore become a fruitful entry point of thinking about issues of gender beyond an unrestricted linguistic constructivism that marginalizes materiality and traditional accounts of natural kinds that marginalize malleability.

The interactive character of material kinds contrasts with the tense relation between naturalness and interactivity in the literature on natural kinds. Hacking (1995) famously distinguished interactive human kinds and indifferent natural kinds. Paradigmatic examples of natural kinds—think of gold, tiger, and water—are indifferent to our classifications of them. They don’t know and don’t care how we classify them. In contrast, paradigmatic examples of human kinds—think of depression, homosexuality, and race—are far from indifferent. Humans are often aware of how they are being classified as well as embedded in institutional practices that respond to classifications, creating feedback loops between classificatory and material practices and turning human kinds into moving classificatory targets in constant flux. As Khalidi (2010) has argued, however, interactive processes are by no means restricted to the domain of human kinds and also affect many other kinds such as domesticated animals that have been shaped by our ways of classifying them. He therefore concludes that “Hacking's identification of interactive kinds renders it difficult to draw a clear boundary between kinds usually regarded as real and those commonly treated as artifactual or non-real” (2010, 336).

Several authors have aimed to reform the notion of natural kind to make space for interactive processes that focus on the dynamic character of classificatory practices (Reydon, 2015; Kendig 2015). Shifting attention towards the interactivity of material kinds is very much in the spirit of these suggestions while avoiding their tensions with the tradition of natural kinds that has often been motivated and even defined through alleged independence from human action. In contrast to these tensions in the tradition of natural kinds, a focus on material kinds makes interactions between classificatory and material practices an unproblematic area of inquiry that opens up space for specifying forms of interactivity along different grades, dimensions, and scales.

### Purpose sensitivity

One important virtue of a framework of material kinds is that “materiality” disrupts the association between realism and freedom from human purposes. Materials are both straightforwardly real and purpose-oriented. While the tradition of natural kinds positions reality in contrast to human purposes, much of the new materialist literature is focused on thinking realism and purposive-sensitivity together as most clearly expressed in Barad’s (2007) “agential realism”. Recognizing material kinds is not about the metaphysical ideal of an interest-independent description but rather about taking material realities seriously in purpose-driven (linguistic and non-linguistic) practices.

This shift in emphasis is especially crucial for addressing socially relevant but contested categories in science such as *biodiversity, ethnicity, disability, genetically modified organism, gender, intelligence, invasive species, sexual orientation, pandemic, poverty, race, refugee,* or *sustainability.* All of these categories can be described as “natural” or “non-natural” depending on the account of natural kinds that is employed. They appear “natural” in the sense that they reflect material structures that can be empirically investigated but “non-natural” in the sense that their boundaries respond to human interests and purposes. Historically, the tradition of natural kinds simply marginalized such unruly categories by focusing unsuspecting categories in the natural sciences—*gold, tiger, water,* etc.—while contrasting them with obviously arbitrary categories such as *animals born on a Tuesday*.

While more recent attempts to reform the tradition of natural kinds often includes purpose sensitive kinds, they risk creating misunderstanding with traditional accounts of natural kinds as “carving nature at its joints” independent of human aims (Brigandt, 2022). For example, applying natural kind frameworks to contested categories from *biodiversity* and *refugee* comes with the risk of a depoliticizing effect by highlighting questions such as predictability or property clustering while leaving processes of social and political negotiation of categories as a secondary thought. An account of material kinds creates space for addressing the social and political negotiation of kinds without reducing it to processes of linguistic construction.

## Material Kinds in Action

The shift from “naturalness” to “materiality” creates a novel and more inclusive entry point for philosophy of classification by moving away from demarcation disputes about the natural/non-natural boundary and instead allowing explorations of the restricted malleability of material kinds along multiple dimensions. To show how such a shift from natural to material kinds makes a difference in practice, let us briefly return to Ludwig’s (2018) discussion of ethnobiology. For example, Ludwig discusses Bulmer’s (1967) classical study of cassowaries that are not considered birds (*yakt*) by the Indigenous Kalam community in Papua New Guinea. Instead, Kalam classifications reflect a complex system of normative and spiritual practices that put cassowaries closer to humans than birds. For example, a hunting taboo applies to cassowaries. Killing a cassowary is spiritually analogous to killing a human that requires a blunt weapon without spilling blood. Both killing cassowaries and humans has complex spiritual ramifications and requires ritual practices such as avoidance of certain crops.

The tradition of natural kinds suggests a quick dismissal of *yakt* as not reflecting joints in nature but contingent cultural and ritual conventions. Dismissing *yakt* as a non-natural kind, however, fails to provide an entry point for engaging more seriously with this classificatory practice and suggests lumping it together with arbitrary categories such as “animals born on a Tuesday”. In contrast, an account of material kinds provides an entry point for multidimensional exploration of *yakt* as a material kind that is characterized through different features of restricted malleability. First, there is indeed a high *degree* of malleability through cultural and ritual forming but the exclusion of cassowaries from the bird ethnotaxon *yakt* is not arbitrary. There are not only cultural *dimensions* but cassowaries also differ from other birds anatomically and morphologically—e.g. they are much larger than other birds (roughly the size of humans) and have different leg bones (more similar to human legs). These different dimensions are causally connected along different *scales*—e.g. the morphological features affect behavior (e.g. cassowaries do not fly) which affect the ecological roles (e.g. terrestrial habitat affects position in food cycles) of cassowaries. A lot of research questions about the *interaction* between these features and classificatory practices emerge. For example, the exclusion of cassowary from *yakt* may be hypothesized as adaptive in preventing overhunting of these easy (large and terrestrial) targets and therefore as a crucial part of Indigenous expertise for the *purpose* of sustainable engagement with a local ecosystem. The question whether *yakt* is a natural kind is a non-starter and suggests quick dismissal. Exploring *yakt* as a material kind along the five features of restricted malleability is fruitful and can actually connect philosophical analysis with empirical research that aims to make sense of local classificatory practices.

Consider another case study such as Ludwig’s (2018) discussion of soil types. Indigenous classifications of soils are often driven by distinctly local concerns about morphological soil patterns in a given environment or their suitability for local practices of agricultural production. While distinctions between soil types are often deeply pragmatic and strikingly local, they are also commonly based clusters of properties that make them projectible for relevant—e.g. agricultural—practices. From the perspective of natural kinds, soil types are problematic entities that get stuck in demarcation disputes. According to some current accounts such as Slater’s (2015) stable property clusters, local soil types will often qualify as natural kinds while other accounts such as Franklin-Hall’s (2015) categorical bottlenecks will exclude them (see Ludwig, 2018).

From the perspective of material kinds, soil do not raise demarcation problems as they are straightforward and unproblematic examples of material kinds. That does not mean that there is nothing interesting to say about them as the structure of soil types can be explored through a multidimensional account of restricted malleability that highlights (1) degrees, (2) dimensions, (3) scales, (4) interactivity, (5) and purpose sensitivity. First, using an account of material kinds as an entrance point highlights that soil types come with a relatively high *degree* of malleability as academic and non-academic communities classify soils in remarkably different ways (Krasilnikov and Tabor, 2003). In this sense, soil types exhibit a higher degree of malleability than many classical cases of natural kinds in ethnobiology such as *jaguars* which are recognized as a distinct kind by Indigenous communities and academic researchers alike. This is not to say that “anything goes” and there are indeed also cases of convergence between academic and non-academic soil classifications (Barrera-Bassols & Zinck, 2003). On a spectrum of malleability, soil types fall in-between intersubjectively recognized “joints in nature” and “arbitrary conventions” that have received most of the philosophical attention but only mark idealized end points on a spectrum of different degrees of malleability.

Second, this graduality can be partly understood in terms of different *dimensions* of malleability in ethnopedological studies of soil classification. Some dimensions such as soil morphology are recognized by diverse (academic and non-academic) actors and therefore lead to cross-culturally converging classificatory patterns. Other dimensions shape soil classifications of specific actors: for example, a soil scientist may focus on structural dimensions of chemical composition that are not addressed by a local community while the community may consider functional dimensions of growing conditions for local crops and agricultural practices that are unknown to scientists. As a result of this partial overlap in attention to dimensions, classifications of soils show both divergence and convergence.

Third, different dimensions of soil classification are related to different *scales*—e.g. along chemical, morphological, and ecological levels of organization—that do not only receive different attention by different classificatory systems but are also connected with each other. A local community, for example, may not explicitly reflect on the chemical level but still carve up soil types partly along chemical properties because they affect morphological properties of soil and growing conditions of crops. One key result is that different actors can classify soil along different scales but still converge through the links between scales such as chemical composition affecting soil morphology which is in turn used as an indicator for growing conditions in a local agricultural practice.

Fourth, the case of soil seems to involve a lower level of *interactivity* than many human kinds that are characterized by “looping effects” (Hacking, 1995) in the sense that humans often become aware of how they are being classified and respond to this classification. Soils do not know or care how they are classified. This does not mean that soil types are entirely non-interactive. For example, human actions commonly cause soil erosion (e.g. through climate change, deforestation, monocropping, pesticides, fertilizers) that transforms the material structure of soils which in turn can feed back into the way humans classify soil. At the same time, classificatory practices can also respond to the emergence of novel practices—e.g. in agroecology and regenerative agriculture—that aim to mitigate effects of soil erosion, thereby creating complex interactive dynamics between material interventions into soil and classificatory practices. Exploring these dynamics would constitute a relevant research project that could contribute to understanding classificatory practices far beyond the generic question whether soil types are deserving of the title “natural kind.”

Finally, different degrees, dimensions, scales, and interactions showcase the *purpose-sensitivity* of classificatory practices. Indigenous soil classifications are troubling for many accounts of natural kinds because they are often shaped by very specific local practices such as *milpa*, an Indigenous Mesoamerican crop rotation system that focuses classificatory attention to the suitability of soil for rotating maize, beans, and squash. At the same time, it would be a mistake to assume that Indigenous soil classifications have a single-purpose character that allows direct translation into categories such as *soil suitable for maize, soil suitable for beans,* and *soil suitable for squash*. Instead, Indigenous soil classifications usually have a more multi-purpose character that can reflect suitability of diverse crops but also other dimensions such as soil preparation (e.g. tillage or drainage conditions) or properties beyond agriculture (e.g. naturally occurring biodiversity, geographic features). Exploring the links between different dimensions and scales of materiality can illuminate this interplay beyond a simple dichotomy of natural “joints in nature” that are recognized independently of any purposes and superficial “single purpose” categories that would not be able to explain the suitability of Indigenous soil categories for a variety of practices.

The case of soil types illustrates how a move from naturalness to materiality provides an entry point for exploring classificatory practices with epistemic and non-epistemic nuance. Rather than focusing on the contested status of soil types as natural kinds, their uncontroversial status as material kinds provides a fruitful ground for specifying their restricted malleability as gradual, multi-dimensional, scalable, interactive, and purpose sensitive. Responding to Conix and Chi’s (2021) demand for a general entry point, an account of material kinds provides a nuanced toolbox for analysis while an account of natural kinds leads back into demarcation disputes.

## Reimagining Philosophy of Scientific Classification

Conceptual change in philosophy and science involves reinterpretations of many epistemic core concepts in science such as *explanation, model*, *objectivity,* or *probability* (Daston & Galison, 2007)*.* Sometimes, it is more productive to reform existing conceptual resources than to abandon them altogether (Chang, 2011) and it seems plausible to interpret the tradition of natural kinds through this lens of conceptual change. While *natural kind* has been associated with a range of problematic assumptions about essences, mind-independence, and monism, much of the more recent literature has aimed to reform *natural kind* to make the concept compatible with the plurality of scientific classifications and its interactions with diverse epistemic and non-epistemic aims in scientific practice.

While it is certainly possible to conserve concepts through gradual reform, the implication should not be an extreme conservatism that never allows for more substantial reframing. Sometimes it is better to move on. In the case of *natural kind*, there has been growing frustration in philosophy of science (Hacking, 2007; Chakravartty, 2017; Ludwig, 2018; Brigandt, 2022) but a straightforward eliminativism also risks eliminating an important entry point for engaging with scientific classification. The aim of this article has been to argue for a substitutionism that navigates between reformism and eliminativism by shifting from natural to material kinds.

The success of this substitutionist strategy depends on it preserving important insights from the tradition of natural kinds while providing opportunities to move beyond its limitations. One important area of continuity between natural and material kinds is a broad realism that contrasts with poststructuralist philosophies of classification and the risk of misframing categories as mere linguistic constructions. Another area of continuity is that the proposed account of material kinds allows the identification of more restricted subclasses of material kinds by specifying malleability through different (1) degrees, (2) dimensions, (3) scales, (4) interactivity, (5) and purpose sensitivity. Such a specification allows for a fine-grained analysis that incorporates insights from the tradition of natural kinds. For example, some material kinds have a relatively low degree of malleability because they are distinguished by particularly stable property clustering and/or projectibility. Other kinds are restricted through their causal unification across scales—e.g. when chemical or genetic or neural structures can be identified as causes of a wide range of observable macroproperties.

An account of material kinds in terms of restricted malleability is also in continuity with the tradition of natural kinds through its broad realism and ability to acknowledge more restricted subclasses of kinds (e.g. in terms of property clusters or projectibility) that are relevant in scientific practice. At the same time, it departs from the tradition of natural kinds through its inclusivity in the sense that it includes many scientific and ordinary kinds (say *biodiversity, class, gender, planet, table, vegetable*) whose status as natural kinds is doubtful. Materiality is “easy” (Thomasson, 2014), especially when understood through feminist materialism that incorporates insights from historical materialism and therefore includes not only biological bodies but also social structures and practices as paradigmatically material and pointing beyond issues of linguistic construction.

The inclusivity of restricted malleability circumvents demarcation debates that have dominated much of the tradition and of recent debates about natural kinds. Rather than having to decide between the many different demarcation criteria that have been proposed in the literature (homeostatic property clusters, stable property clusters, projectibility, nodes in causal networks, grounded functionality, categorical bottlenecks, and so on), an account of material kinds can explore how these criteria identify relevant subsets of material kinds without prioritizing any of them as a general demarcation criterion. As such, an account of restricted malleability provides an inclusive entry point that still allows for exploration of more restricting features that have been highlighted in the tradition of natural kinds. At the same time, an account of material kinds leaves room for human aims that often remain marginalized in debates about natural kinds (Brigandt, 2022) and have often led to superficial engagements with social and political dimensions of classificatory practices. In this sense, restricted malleability provides an inclusive entry point for exploring the complex reality of classificatory practises that has become an increasing concern in philosophy of science (Kendig, 2015; Reydon, 2015) but tends to be distorted through an intellectual tradition that downplays human aims through the metaphysics of naturalness.

An account of material kinds preserves important insights of the tradition of natural kinds while avoiding much of its baggage. Still, one may wonder whether it is actually feasible to instigate conceptual change from “naturalness” to “materiality”. This worry can be further articulated through recent debates about an “implementation challenge” (Queloz and Bieber, 2022; Jorem, 2021; Isaac & Koch, 2022) of conceptual engineering—philosophers have neither the authority nor power to become the language police of scientific or public discourse. From a naturalist perspective that treats philosophy and science as continuous, however, the implementation challenge rests on a misleading distinction: Conceptual change is ubiquitous in scientific practice and if philosophers of science are part of these practices (rather than policing them from the outside), there is nothing suspicious about proposing and testing new conceptual tools. Conceptual negotiation and innovation is just daily practice in academic discourse. Of course, the usefulness of a shift from “naturalness” to “materiality” would still have to prove itself in practice beyond the abstract arguments of this article. However, there are at least some reasons for optimism as the proposed shift does not derive solely from abstract arguments but rather from already successful practices in feminist science studies that use “materiality” to navigate between the shortcomings of the tradition of natural kinds and poststructuralism. It may just be time for philosophers of science to listen, learn, and experiment.
